# Antibacterial activity of some selected medicinal plants of Pakistan

**DOI:** 10.1186/1472-6882-11-52

**Published:** 2011-06-30

**Authors:** Yamin Bibi, Sobia Nisa, Fayyaz M Chaudhary, Muhammad Zia

**Affiliations:** 1 Department of Microbiology, Quaid-i-Azam University Islamabad 45320 Pakistan; 2 Department of Biotechnology, Quaid-i-Azam University Islamabad 45320 Pakistan

## Abstract

**Background:**

Screening of the ethnobotenical plants is a pre-requisite to evaluate their therapeutic potential and it can lead to the isolation of new bioactive compounds.

**Methods:**

The crude extracts and fractions of six medicinal important plants (*Arisaema flavum*, *Debregeasia salicifolia*, *Carissa opaca*, *Pistacia integerrima*, *Aesculus indica*, and *Toona ciliata*) were tested against three Gram positive and two Gram negative ATCC bacterial species using the agar well diffusion method.

**Results:**

The crude extract of *P. integerrima *and *A. indica *were active against all tested bacterial strains (12-23 mm zone of inhibition). Other four plant's crude extracts (*Arisaema flavum*, *Debregeasia salicifolia*, *Carissa opaca*, and *Toona ciliata*) were active against different bacterial strains. The crude extracts showed varying level of bactericidal activity. The aqueous fractions of *A. indica *and *P. integerrima *crude extract showed maximum activity (19.66 and 16 mm, respectively) against *B. subtilis*, while the chloroform fractions of *T. ciliata *and *D. salicifolia *presented good antibacterial activities (13-17 mm zone of inhibition) against all the bacterial cultures tested.

**Conclusion:**

The methanol fraction of *Pistacia integerrima*, chloroform fractions of *Debregeasia salicifolia *&*Toona ciliata *and aqueous fraction of *Aesculus indica *are suitable candidates for the development of novel antibacterial compounds.

## Background

During the last decades, there is increasing interest to unlock the secrets of ancient herbal remedies. For this purpose, various strategies have been developed e.g., biological screening, isolation as well as clinical trials for a variety of plants. Based on the screening methodologies, the therapeutic values of many herbal medicines have already been established. Although, herbal medicines are obtained from natural sources, and considered as safe for human beings. On the contrary, they would have some adverse effects due to the presence of other active ingredients [1].

In the worldwide as well as in the developing countries, the most human died due to infectious bacterial diseases [2]. The bacterial organisms including Gram positive and Gram negative like different species of *Bacillus*, *Staphylococcus, Salmonella *and *Pseudomonas *are the main source to cause severe infections in humans. Because these organisms have the ability to survive in harsh condition due to their multiple environmental habitats [3]. The synthetic antibiotics have the following limitation: Firstly, these are costly and are out of range from the patient belonging to developing countries. Secondly, with the passage of time microorganism develop resistance against antibiotics. Therefore, after some time these antibiotics are not effective against the microbes [4,5]. Furthermore, the antibiotics may be associated with adverse effects on the host, including hypersensitivity, immune suppression, and also allergic reactions. On the other hand, natural products have got incredible success in serving as a guidepost for new antibacterial drug discovery. Moreover, antibiotics obtained in this way have biological friendliness nature [6,7]. As is well known that the bioactive plant extracts is a promising source of majority of drugs [8]. For example, Quinine (*Cinchona*) and berberine (*Berberis*) are the antibiotics obtained from plants which are highly effective against microbes (*Staphylococcus aureus, Escherichia coli) *[9].

In Pakistan, a vast diversity of bioactive plants grown naturally. In the present study, we have investigated the bioactivity of following six naturally growing plants: *Aesculus indica *Linn., *Arisaema flavum*, *Debregeasia salicifolia, Pistacia integerrima *Stew. ex Brand, *Toona ciliate, Carissa opaca*. Their distribution, traditional use and properties [10-30] are described in table 1.

**Table 1:** Summary of plants, parts used and extraction

Plant	Family	Local name	Occurrence in Pakistan/Collection site	Traditional use	Properties	Part used (Kg)	Method of extraction	Extract obtained (g)
*Aesculus indica*	Sapindaceae	Jawaz	northern western Himalayas/Lower Barian Taien valley	Rheumatism, skin diseases, vein complications [10,11]	antioxidant and cell mediated immune response [11,12]	Leaves (1.5)	Cold maceration	250

*Arisaema flavum*	Araceae	Marjarai	sub-alpine regions/Kaghan valley	Snake bite, scorpion sting [13,14]	Anti-proliferative activity against J774 and P388D1 murine cancer cell lines [15]	Rhizome (5)	Soxhelt extraction	250

*Debregeasia salicifolia*	Urticaceae	Ajlai, Chewr	in Swat, salt range, Murree hills Peer Sohava Islamabad	Urinary complaints	Anti-proliferative property against MCF-7 breast cancer cell line [16].	Stem (4)	Cold maceration	200

*Pistacia integerrima*	Anacardiaceae	Villanay	Peer Sohava Islamabad	Cough, phthisis, asthma, dysentery [17]	Hypouricemic, analgesic, anti-inflammatory, antioxidant [18-20]. Antioxidant, radical scavenging, and xanthine oxidase inhibitory activities [21].	Stem (2)	Cold maceration	400

*Toona ciliata*	Meliaceae	Mahanim	QAU campus Islamabad	Astringent, ulcer, fever, rheumatism, chronic dysentery [22,23]	Antibacterial, antifungal, and analgesic [24-26].	Leaves (4)	Cold maceration	200

*Carissa opaca*	Apocynaceae	Granda	Peer Sohava Islamabad	Jaundice, hepatitis, cough, fever, diarrhea [27-29]	antioxidant activity [30]	Leaves (6)	Cold maceration	200

Herein we demonstrate, antibacterial screening of crude extracts of these six medicinally important plant species, and their extract fractions were carried out by agar well diffusion method. The biological activity of plant extracts was tested against Gram positive and Gram negative clinical isolates from American Type Culture Collection (ATCC).

## Methods

### Collection and extraction

The plants material was collected from different geographical regions of Pakistan. *Arisaema flavum *was collected from Kaghan valley; *Debregeasia salicifolia*, *Carissa opaca*, and *Pistacia integerrima *were collected from Peer Sohava Islamabad, *Aesculus indica *(Linn.) was collected from Lower Barian Taien valley; while *Toona ciliata was *collected from Quaid-i-Azam University campus Islamabad. The collected plants were identified by Dr. Mir Ajab Khan, Department of Plant Sciences, Quaid-i-Azam University Pakistan after examination of the specimens already presented in herbarium.

Each plant material was thoroughly washed under running tap water and dried under shade. The dried material of *Arisaema flavum *rhizome (5 Kg), *Debregeasia salicifolia *stem (4 Kg)*, Pistacia integerrima *stem (2 Kg)*, Aesculus indica *leaves (1.5 kg), *Carissa opaca *leaves (6 kg) and *Toona ciliate *leaves (4 kg) was ground to powder form for extraction. Soxhelt extraction technique was used for extraction from *Arisaema flavum*. Soxhelt apparatus was used to carry out the extraction. For this purpose, plant material was divided into two portions. Each portion was extracted with 200 ml methanol. Quantity of the crude extract obtained was 250 g. While, other five plants (*Debregeasia salicifolia, Pistacia integerrima*, *Aesculus indica*, *Carissa opaca *and *Toona ciliata*) were subjected to cold maceration technique for extraction. Powdered material of each plant was soaked in methanol separately at room temperature. After seven days, the extract was filtered under vacuum through Whatman filter paper No. 1. The residue was again dipped in methanol for additional seven days and filtered thereafter. The filtrates were combined and methanol was evaporated under vacuum, using rotary evaporator (Buchi Rotavapor R-200) at 45°C. The quantities of extracts obtained from *Debregeasia salicifolia, Pistacia integerrima*, *Aesculus indica*, *Carissa opaca*, and *Toona ciliata *were 200, 400, 250, 200, and 200 g, respectively (Table 1).

### Fractionation

**Figure 1:**
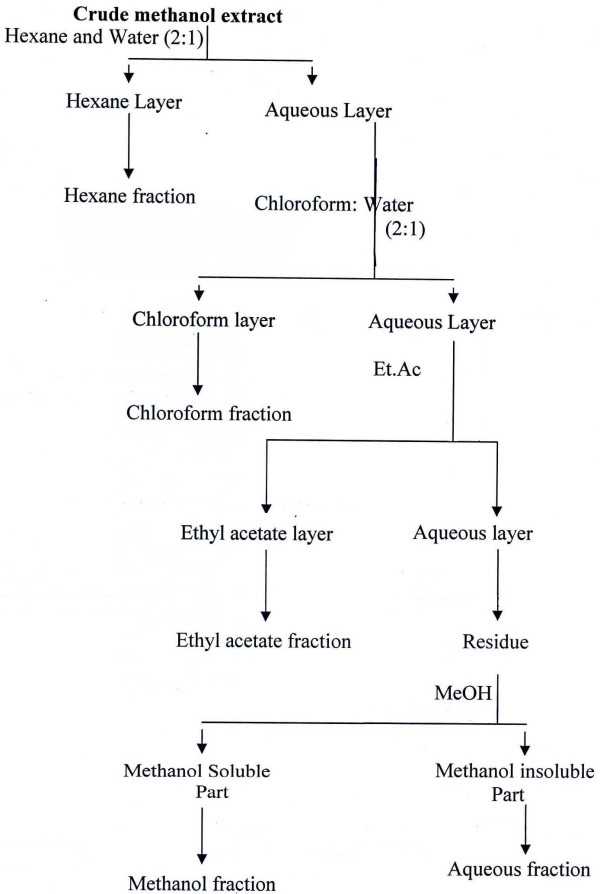
**Fractionation scheme of plants crude extracts**.

Fractionation was carried out by suspending each extract in 250 mL water separately and partitioning with different organic solvents (hexane, chloroform, ethyl acetate, and methanol) in order of increasing polarity by using separating funnel (Figure 1). All the five fractions of each plant extract were dried by evaporating respective solvent using rotary evaporator. All extracts were stored at 4°C till further analysis.

### Antibacterial activity

The agar well diffusion method was performed to exploit antibacterial potential of used extracts [8]. Each extract (200 mg) was dissolved in 10 mL of 99.9% dimethyl sulfoxide (DMSO) (Sigma-Aldrich USA) to get 20 mg/mL concentration. Cefotaxime (2 mg/mL) in DMSO was prepared as positive control. Pure DMSO (99.9%) was used as negative control.

### Test microorganisms

In the present study, total five bacterial strains were used for assay. Three of them were Gram positive, i.e. *Bacillus subtilis *(ATCC 6633), *Staphylococcus aureus *(ATCC 6538), and *Micrococcus luteus *(ATCC 10240). Out of five bacterial strains, two were Gram negative namely *Salmonella setubal *(ATCC 19196) and *Pseudomonas pickettii *(ATCC 49129). The nutrient broth medium (8 g/L) was prepared by dissolving nutrient broth (Merck) in distilled water. At pH = 7.0, 50 mL of media was dispensed in a 100 mL flask and then autoclaved. The bacterial cultures were inoculated individually and kept at 37°C overnight in a shaker incubator at 150 rpm.

### Turbidity Standard and preparation of inocula

The turbidity standard was prepared by mixing 0.5 ml of BaCl_2 _(0.048 M) in 99.5 mL 0.3 6N H_2_SO_4_.BaSO_4. _The standard was taken in screw cap test tube to compare the turbidity. The bacterial culture of selected strains were grown overnight, and subsequently mixed with physiological saline. Turbidity was corrected by adding sterile saline until a McFarland 0.5 BaSO_4 _turbidity standard 10^8 ^Colony Forming Unit (CFU) per ml was achieved. These inocula were used for seeding of the nutrient agar.

### Procedure

Nutrient agar medium was prepared by suspending nutrient agar (Merk) 20 g/L in distilled water. The pH value of the media was adjusted to 7.0, autoclaved, and allowed to cool up to 45°C. The media was seeded with 10 mL prepared inocula. Subsequently, the seeded medium (75-80 mL) was poured into pre-labeled Petri plates (diameter = 14 cm) and allowed to solidify. Required numbers of wells per plate (six wells for extracts, one for positive and negative control each) were made with 8 mm sterile cork borer. These wells were sealed by pouring 20 μl of liquid nutrient agar medium in each well.

With the help of micropipette, 100 μl of test solution was poured into respective well. Sample of extracts, one positive control (Cefotaxime), and one negative control (DMSO) were applied to each Petri plate. Then the plates were incubated at 37°C. After 24 hr of incubation period, diameter of clear zones around each well was measured, showing no bacterial growth. In this study triplicate plates were prepared for each extract and bacterial strain. The mean zone of inhibition was calculated with standard deviation procedure. The percentage growth inhibition was calculated by,

Where TS, SC and PC represents test sample, solvent control and positive control, respectively.

### Statistical analysis

Analysis of variance (ANOVA) and Least Significant Difference (LSD) test at p < 0.05 was carried out using MSTATC to determine the significance of percentage inhibition values between the extracts against bacterial strains.

## Results and discussion

Here, six medicinal plants used for different remedies by local communities were tested against ATCC bacterial cultures to determine and investigate their antibacterial potential. We observed that the crude methanol extract of *Pistacia integerrima *showed significant antibacterial activities against all the tested bacterial strains. Maximum activity was conferred against *Staphylococcus aureus *(23 mm) while minimum was observed against *Micrococcus luteus *and *Salmonella setubal *with mean inhibition zone diameter 17 and 17.33 mm, respectively (Table 2).

**Table 2:** Zone of inhibition (mm; in diameter) against different bacterial strains by plants extracts

Plant	Extract	Microorganisms used				
		
		*B. subtilis*	*M. luteus*	*S. setubal*	*S. aureus*	*P. pickettii*
*P. integerrima*	Crude	20.33 ± 0.41	17 ± 0	17.33 ± 0.34	23 ± 0.34	19.66 ± 0.05
	
	Hexane	14.6 ± 0.11	13.3 ± 0.05	13.3 ± 0.2	12 ± 0.2	10.3 ± 0.05
	
	Chloroform	13 ± 0	12 ± 0	12 ± 0	11.66 ± 0	12 ± 0
	
	Ethyl acetate	15 ± 0	12.66 ± 0.05	14.3 ± 0.37	13.3 ± 0.2	-
	
	Methanol	-	-	10 ± 0	11.5 ± 0	11 ± 0.5
	
	Aqueous	19.66 ± 0.05	-	-	-	-
	
	Cefotaxime	40 ± 0	33.6 ± 0.05	33.3 ± 0.05	33 ± 0.2	30 ± 0
	
	DMSO	-	-	-	-	-

*T. ciliate*	Crude	11.2 ± 0.06	17 ± 0.41	17.1 ± 0.06	-	12.3 ± 0.01
	
	Hexane	10.6 ± 0.21	-	-	-	-
	
	Chloroform	13.6 ± 0.06	15.8 ± 0.12	14.5 ± 0.04	13 ± 0.08	-
	
	Ethyl acetate	15 ± 0.06	16.2 ± 0.09	-	-	11.2 ± 0.11
	
	Methanol	-	12.7 ± 0.07	-	-	-
	
	Aqueous	-	-	-	-	-
	
	Cefotaxime	35 ± 0.05	36.3 ± 0.06	31.5 ± 0.04	31.5 ± 0.05	24.1 ± 0.04
	
	DMSO	-	-	-	-	-

*C. opaca*	Crude	10.2 ± 0.07	-	-	-	-
	
	Hexane	-	-	-	-	-
	
	Chloroform	11.3 ± 0.03	-	-	10.2 ± 0.05	-
	
	Ethyl acetate	12.3 ± 0.04	-	-	-	-
	
	Methanol	-	-	-	-	-
	
	Aqueous	-	-	-	-	-
	
	Cefotaxime	32.7 ± 0.09	39.2 ± 0.11	33.2 ± 0.08	37.3 ± 0.08	28.1 ± 0.05
	
	DMSO	-	-	-	-	-

*A. indica*	Crude	12 ± 0	14 ± 0.5	13.5 ± 0.5	14.5 ± 1	13 ± 0.5
	
	Hexane	-	-	-	-	-
	
	Chloroform	10.5 ± 0.1	12 ± 0	11.5 ± 0.5	13 ± 0.5	14.5 ± 0.1
	
	Ethyl acetate	12 ± 0.5	**-**	13 ± 0.5	12 ± 0.2	12 ± 0.5
	
	Methanol	10 ± 0.5	11 ± 0.1	10 ± 0.1	11 ± 0.2	10 ± 1
	
	Aqueous	16 ± 1	14 ± 0.5	15 ± 0.5	13 ± 0.5	13 ± 0.2
	
	Cefotaxime	31 ± 0.5	30 ± 0.4	30 ± 0.05	33 ± 0	32 ± 0.01
	
	DMSO	-	-	-	-	-

*A. flavum*	Crude	10.3 ± 0.17	10.6 ± 0.07	10.6 ± 0.05	**-**	13.7 ± 0.05
	
	Hexane	**-**	**-**	**-**	**-**	-
	
	Chloroform	11.2 ± 0.08	12 ± 0	**-**	10.3 ± 0.42	-
	
	Ethyl acetate	-	9.6 ± 0.61	**-**	**-**	-
	
	Methanol	12 ± 0.5	**-**	12.6 ± 0.02	13.6 ± 0.23	-
	
	Aqueous	-	**-**	**-**	**-**	-
	
	Cefotaxime	36 ± 0.05	38.3 ± 0.02	31.3 ± 0.05	38.6 ± 0.02	26.1 ± 0.03
	
	DMSO	-	**-**	**-**	**-**	-

*D. salicifolia*	Crude	14 ± 0.03	**-**	**-**	-	13.2 ± 0.05
	
	Hexane	-	**-**	**-**	-	-
	
	Chloroform	14.1 ± 0.12	15.1 ± 0.14	13.3 ± 0.09	12.1 ± 0.21	17.2 ± 0.07
	
	Ethyl acetate	13.7 ± 0.11	**-**	**-**	12.3 ± 0.13	13.7 ± 0.01
	
	Methanol	-	**-**	**-**	-	-
	
	Aqueous	-	**-**	**-**	11.6 ± 0.08	-
	
	Cefotaxime	36 ± 0.05	38.3 ± 0.02	31.3 ± 0.05	38.6 ± 0.02	26.1 ± 0.03
	
	DMSO	-	**-**	**-**	-	-

± - mean standard deviation of triplicate, (-)- no zone of inhibition observed, Cefotaxime-as positive control, DMSO-as negative control

The fractions of *P. integerrima *extract showed activity to different extent against different bacterial strains. However, *Bacillus subtilis *was found more susceptible among other tested strains as all fractions except methanol fraction exhibited activity against it. This observation contradicts previous findings that *Bacillus subitilis *was found least sensitive among other strains against different plant extracts [31]. *Bacillus subtilis *has also been reported most sensitive among different strains [32]. The aqueous fraction showed maximum inhibition value (19.66 mm) among other fractions against *Bacillus subtilis*. The crude extract and all fractions except aqueous fraction were active against *S. setubal *and *S. aureus*. Crude extract also exhibit remarkable activity against *P. pickettii *with mean zone of inhibition 19.66 mm. *Pistacia integerrima *contains beta sitosterol [33] that has been reported for antimicrobial properties.

The crude extract of *Toona ciliata *leaves was significantly active against all bacterial strains except *Staphylococcus aureus*. Maximum zone of inhibition (17.1 mm) was observed against *Salmonella setubal*. All five fractions were active, but the chloroform fraction was observed as most active fraction. It showed considerable activity against all bacterial strains except *Pseudomonas picketti*. Maximum inhibition zone (15.8 mm) was shown against *Micrococcus leutus *(Table 2). Previous studies indicate the presence of terpenoids and liminoids [34], and sterols [35] in the *Toona ciliata *plant extract that might be responsible for bactericidal activity.

The crude extract of *Carissa opaca *showed least activity against *Bacillus subtilis *(10.2 mm). The extract was inactive against other tested bacterial strains. The chloroform and ethyl acetate fractions also active against *Bacillus subtilis *slightly more than the crude extract, representing zones of inhibition 11.3 and 12.3 mm, respectively (Table 2). *Carissa opaca *extract contain a large portion of hydroxyacetophenone along with other chemical compounds [36] which might be responsible for antibacterial activity. No other fraction was active against any bacterial strain used in the present study.

*Aesculus indica *crude extract showed moderate activity with the range of inhibition zone 12-14.5 mm. Maximum inhibition was observed against *Staphylococcus aureus *and minimum inhibition against *Bacillus subtilis*. Extract also showed good inhibition zone (14 mm) against *Micrococcus luteus*. *Salmonella setubal *and *Pseudomonas picketii *were moderately inhibited showing zone of inhibition 13.5 mm and 13 mm, respectively (Table 2). Hexane fraction of *Aesculus indica *did not show any activity against any tested bacterial strains. The chloroform fraction showed almost same level of activity (10.5-14.5 mm) similar to crude extract against all bacterial strains. Ethyl acetate and methanol fraction showed moderate inhibition, however, aqueous fraction proved most active among all fractions showing maximum inhibition zone 16 mm against *Bacillus subtilis*. Maximum activity by aqueous fraction might be due to presence of compounds which are polar in nature. It is evident from the traditional use of water decoctions to treat ailments [37].

*Arisaema flavum *crude extract was active against all bacterial strains except *Staphylococcus aureus*. Maximum zone of inhibition (13.9 mm) was observed against *Pseudomonas picketti*. An average zone of 10-11 mm was observed against *Micrococcus leutus, Bacillus subtilis*, and *Salmonella Setubal*. Chloroform and methanol fractions of *Arisaema flavum *showed moderate activity against three strains while ethyl acetate fraction showed mild inhibition (9.6 mm) of *Micrococcus luteus*. A lectin isolated from *Arisaema flavum *has exhibited mitogenic and antiproliferative activity against cancer cell lines [15]

The crude methanol extract of *Debregeasia salicifolia *stem was active against two bacterial strains. Zones of inhibition; 14 mm and 13.2 mm; were observed against *Bacillus subtilis *and *Pseudomonas picketti*, respectively. The fractions of *Debregeasia salicifolia *indicated the distribution of active constituents in chloroform and ethyl acetate fraction. Chloroform fraction was most active fraction as it showed considerable activity against all bacterial strains. Maximum zone of inhibition was observed against *Pseudomonas picketti *that is 17.5 mm (Table 2). Distribution of activity in less polar fractions indicates that active compounds of the plant are non polar to slightly polar in nature. Aqueous fraction was mildly active only against *S. aureus *with mean zone of inhibition 11.6 mm. 3β-(trans-cinnamoyloxy)-19α-hydroxy-urs-12-ene, 3β,19α-dihydroxy-urs-12-ene and pomolic acid methyl ester isolated from *Debregeasia salicifolia *have shown antimicrobial properties [38].

**Figure 2:**
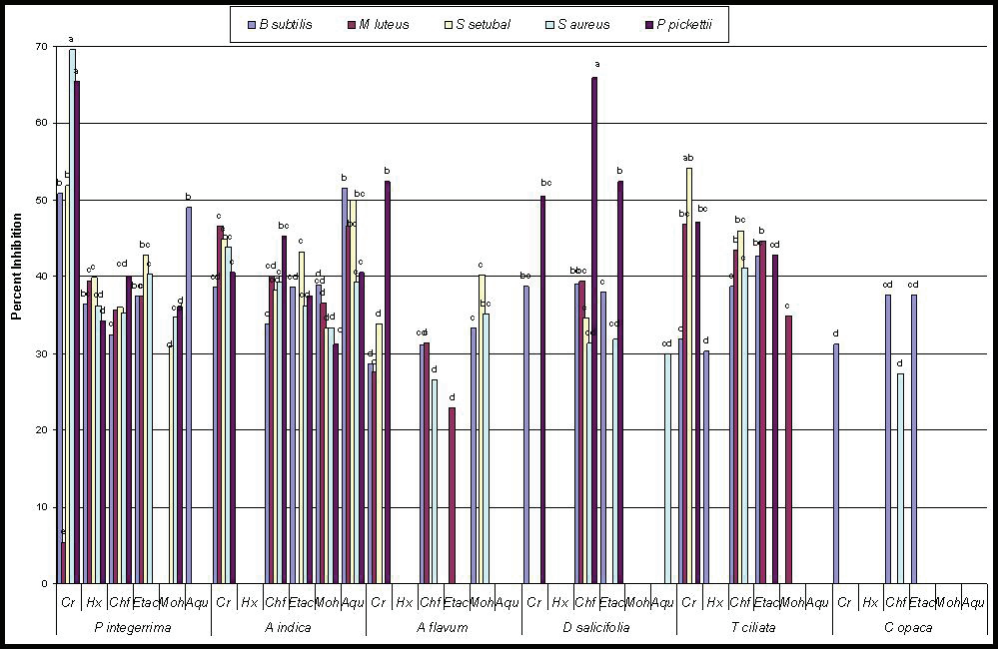
**Comparative percent inhibition of thirty six extracts from six medicinal plants against different bacterial strains**. Alphabets on bars represent LSD values at p < 0.05. Cr = Crude extract, Hx = Hexane fraction, Chf = Chloroform fraction, Etac = Ethyl acetate fraction, Moh = Methanol fraction, Aqu = Aqueous fraction

Figure 2 represents the comparative percent inhibition by all plants extracts. The crude extract of *P. integerrima *showed maximum inhibition of *S. setubal *(69.6%). Same extract also showed 65.5% inhibition of *P. pickettii *while, chloroform fraction of *D. salicifolia *showed good inhibition of *P. pickettii *(65.9%). A number of extracts showed good activities against many strains in a range 40-56% inhibition. Other scientists working in the same filed have also been reported on the variable antibacterial activity by plants extracts and their fractions. Such studies inspired the scientist to identify other bioactive compound through isolation [39-41]. Figure 2 also describes that in few cases crude extract exhibits moderate activity while their respective fractions showed pronounced activity. This might be due to distribution of active component in specific fraction depending upon its solubility nature. While, in few cases the crude extract is more active then fractions. The crude extract of *P. integerrima *exhibited more activity against all tested bacterial strains, as compared to its fractions. In such cases more then one active component might be present in crude extract which distributed into different fractions depending on their solubility. The clinical isolates were inhibited by plant extracts at varying degree. The extracts presenting ≥ 40 inhibition provides a good reason to use these plants for isolation of new antibacterial compound(s).

## Conclusion

In summary, we have described the antibacterial properties of the aqueous fraction of *Pistacia integerrima *and *Aesculus indica*, chloroform fraction of *Debregeasia salicifolia *and *Toona ciliate *against the tested microbes. Therefore, the extracts of these plants were considered as suitable candidates for antibacterial drug discovery. The other extracts showed lower activity which might suggest the lack of bio-active components and/or insufficient quantities in the extract. Based on our findings, we envision that the discovery of novel antibacterial agent from natural sources (plants) will help to minimize the adverse effects of synthetic drugs.

## Competing interests

The authors declare that they have no competing interests.

## Authors' contributions

YB, SN and MZ carried out the experimental part such as extraction, fractionation, preparation of inoculums, antibacterial assay, evaluated the results, and wrote down the manuscript. FMC supervised the work and corrected the manuscript. Authors read and approved the final manuscript.

## Pre-publication history

The pre-publication history for this paper can be accessed here:

http://www.biomedcentral.com/1472-6882/11/52/prepub
